# Comparing the Processing of Music and Language Meaning Using EEG and fMRI Provides Evidence for Similar and Distinct Neural Representations

**DOI:** 10.1371/journal.pone.0002226

**Published:** 2008-05-21

**Authors:** Nikolaus Steinbeis, Stefan Koelsch

**Affiliations:** 1 Max-Planck Institute for Human Cognitive and Brain Research, Leipzig, Germany; 2 Department of Psychology, University of Sussex, Falmer, Brighton, United Kingdom; Yale University, United States of America

## Abstract

Recent demonstrations that music is capable of conveying semantically meaningful information has raised several questions as to what the underlying mechanisms of establishing meaning in music are, and if the meaning of music is represented in comparable fashion to language meaning. This paper presents evidence showing that expressed affect is a primary pathway to music meaning and that meaning in music is represented in a very similar fashion to language meaning. In two experiments using EEG and fMRI, it was shown that single chords varying in harmonic roughness (consonance/dissonance) and thus perceived affect could prime the processing of subsequently presented affective target words, as indicated by an increased N400 and activation of the right middle temporal gyrus (MTG). Most importantly, however, when primed by affective words, single chords incongruous to the preceding affect also elicited an N400 and activated the right posterior STS, an area implicated in processing meaning of a variety of signals (e.g. prosody, voices, motion). This provides an important piece of evidence in support of music meaning being represented in a very similar but also distinct fashion to language meaning: Both elicit an N400, but activate different portions of the right temporal lobe.

## Introduction

The notion that music can convey meaningful information in a fashion similar to language has circulated for some time [1], [2], [3]. Recent neurophysiological studies have put these claims to an empirical test [1]. Using the N400 as electrophysiological marker for processing meaning, it was found that musical pieces were as capable as spoken sentences at priming the processing of subsequently presented target words, as indicated by highly comparable potentials elicited in both conditions. Whereas that study represents a significant advance in providing answers to the phenomenon of meaning in music, further issues remain elusive. This study addresses two of these, the first of which pertains to the underlying mechanisms of how meaning is communicated by music, whereas the second examines if music meaning is represented comparably to language meaning on a neural level.

To provide an answer to the first issue, our first study focussed specifically on the expression of emotions in music as a route to meaning. By using single chords varying in consonance to sound either pleasant or unpleasant, we assessed whether the communicated affect can influence the subsequent processing of verbal emotional meaning. This was investigated by means of an affective priming paradigm using both EEG (Experiment 1a) and fMRI (Experiment 1b), with the hypothesis, that if emotional expression in music is capable of influencing the processing of word meaning, it should become evident by an increased N400 to mismatching word targets as well as by the recruitment of neural structures typically dedicated to processing meaning, such as the middle temporal gyrus [1], [4]

The second issue was addressed in a second study using a very similar paradigm but switching the order of chord-word presentation. This time words were the primes, and chords the target. Seeing that studies on semantic priming typically use word targets and only vary the nature of the prime, we felt that investigating the neural processes underlying music-target processing would provide direct insight into the neural representation of music meaning and how this compares to the representation of language meaning. This was also investigated using both EEG (Experiment 2a) and fMRI (Experiment 2b), with the hypothesis, that should music meaning be represented in similar fashion to language meaning on a neural level, this should become evident by an increased N400 to mismatching chord targets as well as the recruitment of neural structures typically dedicated to processing meaning, such as the middle temporal gyrus [1], [4].

## Methods

### Experiment 1

Participants: Twenty musically trained participants (10 females) with a mean age of 23.6 years and on average approximately 12.3 years of musical training took part in the first study (Experiment 1).

Sixteen musically trained participants (8 females) with a mean age of 24.7 years and on average approximately 15.6 years of musical training took part in the second study (Experiment 2). One subject had to be excluded from the analysis due to having misunderstood the nature of the task.

Informed written consent was obtained from all participants prior to the study. The study in turn was approved by the local ethics committee of the University of Leipzig, and conducted in accordance with the Declaration of Helsinki.

Materials: Word stimuli consisted of a set of 24 pleasant and another of 24 unpleasant words, each of which contained 12 words with either a concrete or an abstract meaning. Chord stimuli consisted of 24 consonant and 24 dissonant chords. Consonant chords were major chords built on either the root or the second inversion. Dissonant chords involved two types of dissonance, one using the first, augmented fourth and seventh key and another using the first, augmented second and augmented fourth key of the scale. Changes in harmonic roughness have been reported to relate to subjectively perceived pleasantness [5], [6]. Both consonant and dissonant chords were played in each of the twelve keys of the Western musical scale, leading to 24 chords in each affective category. On average chords were 800 ms long and a previous rating study had indicated that consonant chords were perceived as significantly more pleasant than dissonant chords (p<0.0001).

**Figure 1:**
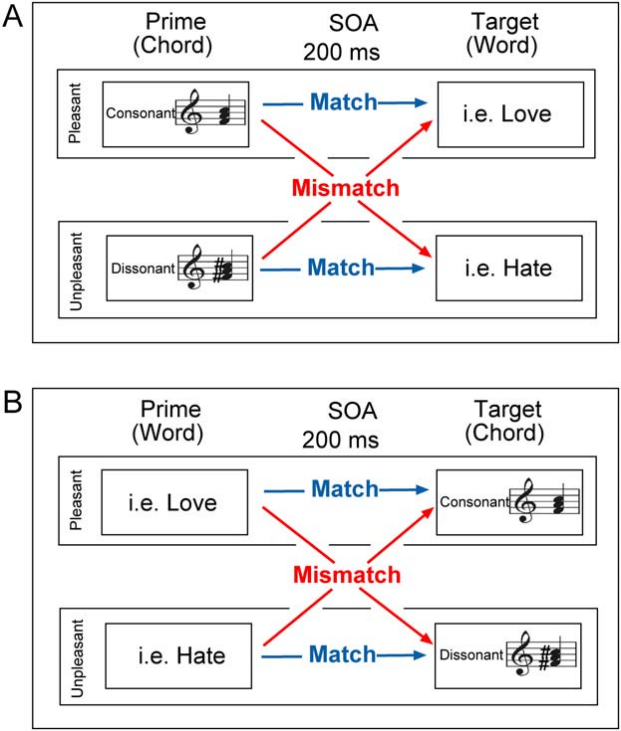
Design of Experiments 1a and 2a (A) and Experiments 2a and 2b (B). In Experiments 1a and 2a chords were presented as primes followed 200 ms after chord onset by target words, which participants were asked to evaluate as fast as possible. In Experiments 1b and 2sb words were presented as primes followed 200 ms after word onset by target chords, which participants were asked to evaluate as fast as possible.

Procedure: In Experiments 1a and 2a chords were played as primes, followed 200 ms after chord onset by the target word, which participants were asked to evaluate as fast as possible (see Figure 1A). Words and chords were matched such that there was a congruent and an incongruent condition, with each chord and each word being presented twice throughout the experiment, once congruently and once incongruently. That way any congruency-related effects would not be attributable to differences in stimuli.

In Experiment 1b and 2b a word was presented for 200 ms followed by the target chord, which participants were asked to evaluate as fast as they could (see Figure 1B). The matching procedure was the same as for Experiment 1a (2a). Seeing that experiments 1a and 1b (and 2a and 2b) were conducted in the same session, the order was counterbalanced across subjects to avoid serial effects.

ERP Recording and Analysis: The EEG was recorded at a sampling rate of 500 Hz from 60 locations of the 10–20 system and referenced to the left mastoid. Data were filtered off-line using a band-pass filter with a frequency range of 0.25–26 Hz (3001 points, finite impulse responses, fir) to eliminate slow drifts and muscular artefacts. All other artefacts (eye movements, head movements) were excluded if the standard deviation of the channel exceeded 25 µV within a gliding window of 800 ms. ERP averages were computed with a 200 ms pre-stimulus baseline and a 1000 ms ERP time window. For statistical analysis, ERPs were analysed by repeated measures Analysis of Variance (ANOVA). Electrodes were grouped into four separate Regions of Interest (ROIs): left anterior, right anterior, left posterior and right posterior. The time windows for statistical analyses were based on visual inspection and previous studies [1], [7].

fMRI Acquistion and Analysis: Imaging was performed on a 3T Trio scanner (Siemens, Erlangen, Germany) equipped with the standard bird-cage head coil. A gradient recalled EPI-sequence was used with TR = 2000 ms and TE = 30 ms. A total of 22 axial slices were collected with a slice thickness of 5 mm and a slice gap of 1 mm. Prior to the functional image acquisition two sets of two-dimensional anatomical images were acquired (T1 Model Driven Fourier Transform (MDEFT) sequence with TR = 1.3 s and TE = 10 ms and an EPI-T1 sequence with the same parameters as the functional run.

Data processing was performed using the software package LIPSIA [8]. Functional data were corrected for motion artefacts and to correct for the temporal offset between slices acquired in one scan, a cubic spline-interpolation was applied. Data were filtered using a temporal highpass filter with a cutoff frequency of 1/128 Hz for baseline correction and a spatial Gaussian filter with 3.768mm full width at half maximum (FWHM) was applied. Functional slices were aligned with a 3D stereotaxic coordinate reference system (acquired for each subjects individually prior to scanning) by means of a rigid linear registration with six degrees of freedom (using three rotational and three translational parameters acquired during the MDEFT and EPI-T1 sequences). The rotational and translational parameters were subsequently transformed by linear scaling to a standard size and the resulting parameters were used to transform the functional slices by using trilinear interpolation (thus, functional slices were aligned with the stereotaxic coordinate system). For the anatomical data, a T-1 weighted, 3-D magnetization-prepared rapid gradient-echo (MP-RAGE) sequence was obtained recording a volume data set with 160 slices and 1 mm slice thickness, which was standardised to the Talairach stereotaxic space [9].

Statistical evaluation was based on a least-squares estimation using the general linear model (GLM) for serially autocorrelated observations [10]. The design matrix was generated using a synthetic hemodynamic response function. The model equation, including the observed data, the design matrix, and the error term, was convolved with a Gaussian kernel, with a dispersion of 4 s FWHM. Contrast images of the differences between the specified conditions were calculated for each subject. The individual contrast images were then entered into a second-level random effects analysis. Subsequently t-scores were transformed into Z-scores. To protect against false-positive activations, only regions with a Z-score >3.09 and with a volume >135 mm (5 voxels) were considered. Given that we had a priori hypotheses about the regions of activations (temporal lobe) this was considered a sufficiently conservative threshold.

Because specific ROIs were specified a priori, further analyses were also conducted over medial temporal structures. ROIs were defined on the functional criterion of local maxima in a contrast map. In order to reduce smearing of activity from different anatomical structures, the local maximum was considered if it was within a radius of 15 mm. Significant maxima were used as the centre of spherical ROIs, which had a radius of 8 mm. To compare condition specific activations, subjectwise contrasts between the critical conditions were calculated, for which normalised Z-scores were averaged over all voxels within a ROI. The resulting mean Z-scores were then subjected to an ANOVA with the factors Prime and Target as repeated measures factors.

## Results

### Experiment 1

#### Behavioural Data

**Figure 2:**
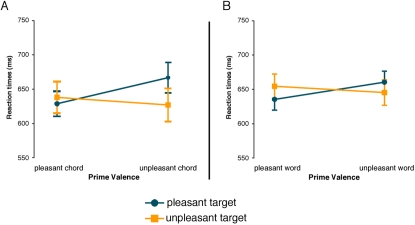
Reaction times (RTs) on correct responses for Experiment 1. The RTs reveal a strong interaction between prime valence and target valence, whereby congruent targets are evaluated significantly faster than incongruent targets. This was the case for incongruent word targets in Experiment 1a (A) as well as incongruent chord targets in Experiment 1b (B).

Experiment 1a:

Analysis of the correct responses showed that congruous target words were evaluated significantly faster than incongruous target words (see Figure 2A). This was indicated by a significant interaction between the factors Prime and Target (F (1,19) = 33,85; p<0.0001). There were no main effects of either prime or target, nor any interactions in the hit rates.

Experiment 1b:

Analysis of the correct responses yielded a significant interaction between Prime and Target (F (1,19) = 26,77; p<0.0001) showing that congruous target chords were evaluated significantly faster than incongruous target chords (see Figure 2B). Again, there were no main effects of either prime or target, nor any interactions in the hit rates.

#### ERP data

**Figure 3:**
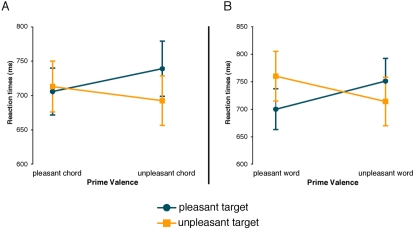
ERPs in response to word targets (A) and chord targets (B). Word targets incongruous with the expressed affect of the preceding chord elicited an increased N400 between 300–500 ms distributed broadly over the scalp (A) with a centro-parietal maximum. Chord targets incongruous with the expressed affect of the preceding word elicited an increased N400 between 200–400 ms distributed broadly over the scalp (B) with a fronto-central maximum.

Experiment 1a:

Analysis of the ERPs, time-locked to the correctly evaluated target words (which were either congruous or incongruous with the preceding chord prime), revealed an increased negativity between 300–500 ms distributed broadly over the scalp in response to incongruous targets (Figure 3A). This was indicated by a significant interaction between factors Prime and Target (F (1,19) = 6,72; p<0.05). There were no main effects of either prime or target.

Experiment 1b:

Analysis of the ERPs, time-locked to the correctly evaluated target chords (which were either congruous or incongruous with the preceding word prime), revealed an increased negativity between 200–400 ms distributed broadly over the scalp in response to incongruous targets (Figure 3B). This was indicated by a significant interaction between factors Prime and Target (F (1,19) = 20,23; p<0.001). There were no main effects of either prime or target.

These data indicate two things. Firstly the affect expressed by single musical features seems to be capable of influencing subsequent word processing at a semantic level, which suggests, that the expression of emotions in music is processed as meaningful by listeners. Secondly, it appears as if the meaning of musical signals is processed in a very similar fashion to meaning contained by language. Particularly the ERP data indicate that musical signals, when incongruous to the prevailing meaning of the context, can elicit an N400, an ERP which has been taken to reflect the processing of meaning [11], [12]. The differences in latency between the N400 elicited by words and chords can be explained by differences in modality of presentation (auditory vs visual), as previous studies have shown earlier N400 onset when targets were presented auditorily compared to visually [13]. To obtain a more definitive answer to how comparable the representation of meaning in music and language is, an fMRI experiment was conducted using the same stimulus as in the ERP experiments.

### Experiment 2

#### Behavioural data

**Figure 4:**
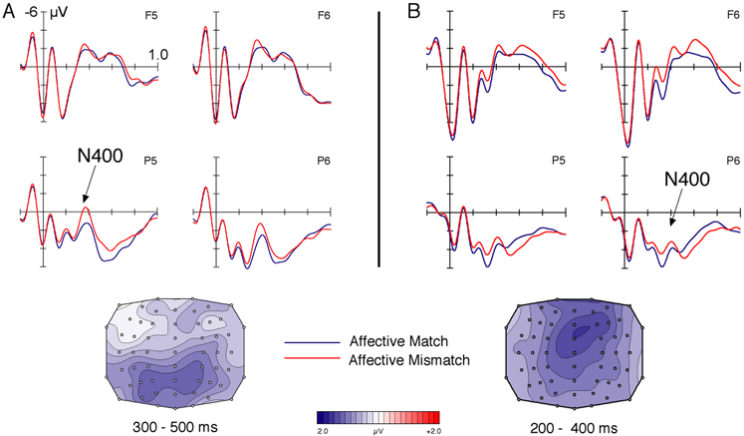
Reaction times on correct responses for Experiment 2. The RTs reveal a strong interaction between prime valence and target valence, whereby congruent targets are evaluated significantly faster than incongruent targets. This was the case for incongruent word targets in Experiment 2a (A) as well as incongruent chord targets in Experiment 2b (B).

Experiment 2a:

Analysis of the correct responses showed that congruous target words were evaluated significantly faster than incongruous target words (see Figure 4A). This was indicated by a significant interaction between the factors Prime and Target (F (1,14) = 23,12; p<0.001). There were no main effects of either prime or target, nor any interactions in the hit rates.

Experiment 2b:

Analysis of the correct responses yielded a significant interaction between Prime and Target (F (1,19) = 21,32; p<0.001) showing that congruous target chords were evaluated significantly faster than incongruous target chords (see Figure 4B). Again, there were no main effects of either prime or target, nor any interactions in the hit rates.

#### fMRI data

**Figure 5:**
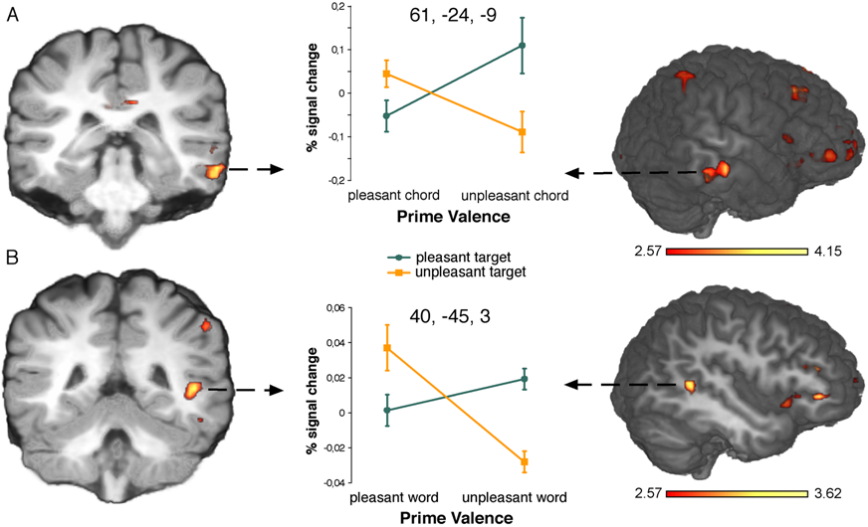
fMRI data in response to word targets (A) and chord targets (B) when contrasting incongruous against congruous trials. Incongruous word targets activated the right MTG (A), whereas incongruous chord targets activated the right posterior STS (B). Data is displayed at a threshold of p<0.005 (uncorrected) for visual purposes.

Experiment 2a:

The fMRI data revealed that target words incongruous to the preceding affective context activated the right MTG (z-value: 3.70; 61, −24, −9) (see Figure 5A). To assess whether these regions also demonstrate the required interaction between Prime and Target, the signal change for each condition was extracted by means of the ROI analysis and subjected to an ANOVA. There was a significant interaction between Prime and Target in the right MTG (F(1,14) = 14,9; p<0.01).

This region has been implicated strongly in processing semantic aspects of language input and its coordinates are relatively close to others reported recently in source localisations of the N400 elicited by target words which were incongruous to a meaningful context established by a musical piece [1; 45, −37, −3]. Congruous word primes did not elicit greater activation at the threshold set for the analysis.

Experiment 2b:

Chord targets incongruous with the preceding word context elicited activations in the right posterior STS (z-value: 3.32; 40, −45, 3) (see Figure 5B). Again, this activation was in the vicinity of the one reported previously in a source localisation of the N400 [1], albeit located slightly more posteriorly, and more deeply within the sulcus. The signal change for each condition was extracted by means of the ROI analysis subjected to an ANOVA with factors Prime and Target, and revealed a significant interaction for the right posterior STS (F(1,14) = 21,7; p<0.001).

These data demonstrate that processing meaning in language and music appears to be subserved by the temporal cortex, with distinct subregions dedicated to processing either the meaning of language or of music. The findings are discussed with reference to previous evidence on the function of these temporal regions in meaning processing bearing in mind the inherent differences between music and language.

## Discussion

The main finding of this study is that the meaning of music appears to be represented in a comparable fashion to language meaning. Both music and language targets incongruous to the preceding affective context elicited an increased N400, a classic indicator of semantic processing [11], [12]. Whereas previous studies have reported findings suggesting that music can convey meaningful information [1], the present findings are the first to show that a musical signal can elicit a meaning related N400 directly, suggesting that the N400 reflects the processing meaning of a wide range of signals, even those not explicitly semantic in nature. Previous reports of an N400 for musical targets [14]. could be explained with respect to the explicit memory violation under investigation and not the communication of musical meaning (as indeed the authors did). Thus, the present study is the first to report an N400 to musical targets which represent a violation of musical meaning.

The fMRI data demonstrate that the neural structures subserving the processing of meaning in language and music are subtly distinct. Recent reviews of the cortical organisation underlying language processing have highlighted the role of the MTG for the processing of lexico-semantic aspects of the language input [15], [16]. It has been argued that the so-called ventral processing stream is critically involved in mapping perceptual input (paradigmatically sounds for language) to its meaning. Whereas this input undergoes several early perceptual stages, the actual mapping of percept and meaning was argued to occur in the middle temporal gyrus (BA 21 /37), a region which was also presently activated by words which are incongruous to the affect expressed by the preceding chord context. Seeing that this incongruity occurs at a higher level of processing (the valence of a word is something that is contained directly in the meaning of the word itself), the present data suggest that processing such an affective/semantic incongruity draws on neural resources typically dedicated to semantic processing. This confirms some previous data on the ability of music to convey meaning and suggests that expressed affect is an important pathway to establishing musical meaning.

The activation of the right posterior STS by the affectively incongruous chord targets, suggests that processing meaning triggered by a musical stimulus does not occur in identical neural structures as the one triggered by a language stimulus. Instead, the posterior STS has been frequently implicated in processing the meaning of a more general nature and a variety of stimulus types other than language. For instance Belin and colleagues [17] reported activations in the posterior STS bilaterally (46, −44, 6; −62, −40, 10) in response to the perception of human voices, compared to a string of non-vocal environmental sounds. Others [18] reported activations of the right superior temporal sulcus (62, −30, 6) in response to emotional (angry) prosody as opposed to neutral prosody. In addition a recent review article on the STS [19] discusses its role in processing the meaning of biologically relevant signals (i.e. eye movements, body movements, lip and mouth movements). All these stimulus types contain and communicate potentially meaningful information (e.g. voices the presence of a conspecific friend or foe, prosody the emotional state of another agent, biological movement the mental states and intentions of another agent), which the STS appears to be sensitive to. The nature of communication of the presently employed musical signal indicates valence, which is in itself a highly salient and also biologically relevant event. We argue that the meaning of emotional musical stimuli is processed not as a specific semantic utterance, but as a signal of more general significance and therefore distinct to the type of meaning expressed by language stimuli. Unlike a language stimulus, the affective meaning of a chord does not have to be mapped onto anything else but is already contained directly in the stimulus itself.

It may be argued, that the effects reported in the present study may not be specific to processing the meaning of music or language, but results from the more general processing of emotional valence. Whereas this account is theoretically plausible, recent data has shown that emotional conflict arising out of pictures and words engages the amygdala, prefrontal regions as well as the rostral anterior cingulate cortex and not temporal regions [20]. This suggests that the involvement of temporal regions may indeed reflect the processing of aspects meaningful specifically to language and music.

Our subjects were musically trained, and it may be that music may achieve its meaning only after a significant amount of being actively exposed to it. However, this issue requires further investigation since the degree of musicianship is also highly correlated with the amount of time spent listening to music.

The present findings suggest that the meaning conveyed by music is processed comparably (N400 and temporal lobe) but not identically (distinct neural loci) to language meaning. Whereas this study used single chords to test this hypothesis (something dictated by using EEG and event-related potentials), it remains an open issue whether the same neural structures dedicated to processing meaning incongruity in music are also responsible for the meaning arising out of large scale musical structures and pieces. This spells out a need for increased efforts in creative experimental design to be able to address this issue.

In conclusion, the present data show that emotion is a primary pathway to establishing meaning in music, something that has hitherto only been assumed [1], [21], [2], but never been directly tested. Both the ERP and fMRI data suggest that semantic information at the word level can be strongly influenced by affect expressed by music. In turn it appears as if the meaning of music is also represented in a similar fashion to language meaning, as indicated by an N400 in response to affectively incongruous chord targets as well as the engagement of neural structures dedicated to processing meaning on a general level. However there are important differences between the nature of meaning in music and language, which are also reflected in the present findings. Whereas language meaning entails mapping the analysis of perceptual input on knowledge contained in a so-called lexicon (memory for meaning of sounds), in the present study no such referential mapping takes place in musical meaning. Instead music is analysed similarly to other types of signals meaningful by virtue of their overall and often specifically biological significance, something which may in the present case be a function of musical expertise and a history of interaction with musical stimuli. The present data do not speak on a specific locus of processing music meaning, much rather it appears as if meaning anything other than that expressed by language appears to be processed in a domain-general fashion, which is dedicated to actions, voices as well as music.

## References

[1] Koelsch S, Kasper E, Sammler D, Schulze K, Gunter T (2004). Music, language and meaning: Brain signatures of semantic processing.. Nat Neurosci.

[2] Sloboda JA (1986). The musical mind..

[3] Swain J (1997). Musical languages..

[4] Maess B, Herrmann CS, Hahne A, Nakamura A, Friederici AD (2006). Localizing the distributed language network responsible for the N400 measured by MEG during auditory sentence processing.. Brain Res Cogn Brain Res.

[5] Van de Geer J, Levelt W, Plomp R (1962). The connotation of musical consonance.. Acta Psychol.

[6] Koelsch S, Fritz T, von Cramon DY, Müller K, Friederici AD (2006). Investigating emotion with music: An fMRI study.. Hum Brain Mapp.

[7] Schirmer A, Kotz S (2003). ERP Evidence for a Sex-Specific Stroop Effect in Emotional Speech.. J Cogn Neurosci.

[8] Lohmann G, Müller K, Bosch V, Mentzel H, Hessler S (2001). Lipsia-A new software system fort he evaluation of functional magnetic resonance images of the human brain. Comput. Med.. Imaging Graph.

[9] Talairach J, Tornoux P (1988). Co-planar Stereotaxic Atlas of the Human Brain: 3-Dimensional Propositional System–an Approach to Cerebral Imaging..

[10] Worsley KJ, Friston KJ (1995). Analysis of fMRI time-series revisited again.. Neuroimage.

[11] Kutas M, Hillyard S (1980). Reading senseless sentences: Brain potentials reflect semantic incongruity.. Science.

[12] Kutas M, Federmeier K (2000). Electrophysiology reveals semantic memory use in language comprehension.. Trends Cogn Sci.

[13] Holcomb P, Neville H (1990). Auditory and visual semantic priming in lexical decision: A comparison using event-related potentials.. Lang Cogn Proc.

[14] Miranda RA Ullman MT (2007). Double dissociation between rules and memory in music: An event-related potential study.. Neuroimage.

[15] Hickok G, Poeppel D (2000). Towards a functional neuroanatomy of speech perception.. Trends Cogn Sci.

[16] Hickok G, Poeppel D (2007). The cortical organization of speech processing.. Nat Rev Neurosci.

[17] Belin P, Zatorre RJ, Lafaille P, Ahad P, Pike B (2000). Voice-selective areas in human auditory cortex.. Nature.

[18] Grandjean D, Sander D, Pourtois G, Schwartz S, Seghier ML (2005). The voices of wrath: Brain responses to angry prosody in meaningless speech.. Nat Neurosci.

[19] Allison T, Puce A, McCarthy G (2000). Social perception from visual cues: Role of the STS region.. Trends Cogn Sci.

[20] Etkin A, Egner T, Peraza DM, Kandel ER, Hirsch J (2006). Resolving Emotional Conflict: A Role for the Rostral Anterior Cingulate Cortex in Modulating Activity in the Amygdala.. Neuron.

[21] Koelsch S, Siebel WA (2005). Towards a neural basis of music perception.. Trends Cogn Sci.

