# Correction: Comparing the Processing of Music and Language Meaning Using EEG and fMRI Provides Evidence for Similar and Distinct Neural Representations

**DOI:** 10.1371/annotation/a2cfd2c0-5084-4426-8868-f55ec0ea5434

**Published:** 2008-05-27

**Authors:** Nikolaus Steinbeis, Stefan Koelsch

The order of Figures 3 and 4 is switched.

